# Ambulatory and Laboratory Stress Detection Based on Raw Electrocardiogram Signals Using a Convolutional Neural Network

**DOI:** 10.3390/s19204408

**Published:** 2019-10-11

**Authors:** Hyun-Myung Cho, Heesu Park, Suh-Yeon Dong, Inchan Youn

**Affiliations:** 1 Center for Bionics, Biomedical Research Institute, Korea Institute of Science and Technology, Seoul 02792, Korea; 2 Division of Bio-Medical Science & Technology, KIST School, Korea University of Science and Technology, Daejeon 02792, Korea; 3 Department of Biomedical Science, College of Medicine, Korea University, Seoul 02841, Korea; 4 Department of Information Technology Engineering, Sookmyung Women’s University, Seoul 04310, Korea

**Keywords:** stress detection, electrocardiogram, deep neural network, convolutional neural network

## Abstract

The goals of this study are the suggestion of a better classification method for detecting stressed states based on raw electrocardiogram (ECG) data and a method for training a deep neural network (DNN) with a smaller data set. We suggest an end-to-end architecture to detect stress using raw ECGs. The architecture consists of successive stages that contain convolutional layers. In this study, two kinds of data sets are used to train and validate the model: A driving data set and a mental arithmetic data set, which smaller than the driving data set. We apply a transfer learning method to train a model with a small data set. The proposed model shows better performance, based on receiver operating curves, than conventional methods. Compared with other DNN methods using raw ECGs, the proposed model improves the accuracy from 87.39% to 90.19%. The transfer learning method improves accuracy by 12.01% and 10.06% when 10 s and 60 s of ECG signals, respectively, are used in the model. In conclusion, our model outperforms previous models using raw ECGs from a small data set and, so, we believe that our model can significantly contribute to mobile healthcare for stress management in daily life.

## 1. Introduction

As interest in health care increases, the importance of stress management has grown. As many people are exposed to the stressful environments, they are more likely to suffer from physical and mental disorders. Indeed, stress has been shown to cause diseases such as depression, asthma, and autoimmune diseases [1]. To observe the changes in our body caused by stress, many researchers have focused on physiological signals, such as electrocardiography (ECG) signals and galvanic skin response [2].

When a person receives stress stimulation, his/her autonomic nervous system reacts to the stress, which results in physiological changes [3]. Among the physiological signals, the ECG enables us to observe how our bodies react to stress. An ECG is an electrical signal which is generated by heart activity. An ECG signal has three main components: The P-wave, QRS-complex, and T-wave. Among them, the time-series intervals between successive R peaks are used to calculate the heart rate variability (HRV) [4]. The HRV can be represented by various parameters that are calculated along the time, frequency, and non-linear domains. These HRV parameters have often been used for stress recognition [5,6,7].

With the recent development of mobile sensors for ECG recording, HRV analysis and stress studies have been actively carried out. However, due to the inherent limitations of HRV analysis, which requires sufficient data to observe the variability, the longer the time window of the ECG record for an HRV analysis is, the more accurate the statistical characteristics can be. In other words, it is difficult to perform an HRV analysis with short-term ECG measurements. Some previous research has demonstrated the minimum time window required for an HRV analysis. Camm et al. [7] recommended at least a 5 min ECG measurement to analyze the HRV. Moreover, the R peaks along the ECG time-series must be detected. To do so, computational algorithms based on the Pan-Tompkins algorithm [8] can be used. These algorithms contain preprocessing steps, such as filtering and differential operations, to find the QRS-complex adequately. Namely, the classical HRV approach requires additional preprocessing steps with a limited window length.

To recognize stress states, several previous studies [9,10,11] have reported classical HRV analysis using machine learning algorithms, such as the support vector machine (SVM), k-nearest neighbors (kNN), adaptive boosting (AB), and logistic regression (LR) methods, along with the ECG and the other physiological signals. Other physiological signals include skin conductance (SC) [9,11], respiration [9,11], and skin temperature (ST) [11].

Table 1 shows a comparative summary of previous studies which used conventional machine learning algorithms, as well as DNN models. The column “Window length” refers to the duration of the ECG measurement used to extract the HRV parameters. The column “Performance” indicates the reported accuracy of the classifier mentioned in the column “Classifier”. Most studies used more than one classifier, but only those that gave the best results are shown in Table 1. The study [11] used different stressors to the other studies [9,10], including the stroop color word test (SCWT), mental arithmetic (MA), and counting numbers. These stimuli are considered to be laboratory-environmental stress stimulation. Castaldo et al. [10] designed an experiment where an ECG was acquired on two different days. One was a day when the participants were undergoing a university verbal examination, and the other day was after a vacation. Although these studies (which were performed in a laboratory-controlled environment) showed an accuracy of about 85%, outside of the laboratory the accuracy reached only 80% [10]. These results might indicate the limitations of the stressors used in the laboratory environment and of the conventional machine learning methods. A conventional machine learning method based on HRV features involves not only the preprocessing of the ECG signals, but also feature selection among the HRV parameters.

As the deep neural network (DNN) approach has recently demonstrated outperforming pattern recognition accuracy [15], some studies [12,13,16,17,18,19,20,21] have utilized DNNs for biomedical engineering applications, including heart arrhythmia classification, medical image classification and enhancement, stress detection, and for other medical diagnoses. U-net [21], which consists of a convolutional neural network (CNN), forms an autoencoder architecture using skip-connection between the encoder and decoder. This architecture could be used in medical imaging applications, including augmentation, classification, and detection. Hannun et al. [16] achieved a performance in arrhythmia detection using a deep convolutional architecture that was similar to or exceeding that of cardiologists.

Hwang et al. [12] proposed the DeepECGNet method, which detects stress using an ultra-short-term (10 s) ECG. They used raw ECG signals for the input of the DNN without extracting the HRV parameters. A model was configured with one CNN and two long short-term memory (LSTM) [22] models. They suggested an optimal convolution filter width and pooling window width, respectively, of 0.6 s and 0.8 s. They recommended selecting the proper hyperparameter values for the convolution filter width and pooling window width, both of which are capable of covering the QRS-complex of an ECG. They designed an experiment for inducing mental and physical stress in participants through MA, SCWT, visual stimuli, and cold pressor. There were 20 and 30 participants, separated into two cases. In the two cases, the model [12] reached 87.39% and 73.96% accuracy. Saeed et al. [13] suggested using a multitask convolutional neural network (MT-CNN). Raw physiological signals configured with the heart rate (HR) derived from an ECG and SC are fed to the MT-CNN to detect stress. There was no mention of how long the duration of the ECG measurement was, but only 300 samples of ECG signals were reported. They achieved 0.918 for the area under the receiver operating characteristic curve (AUROC).

Though these studies suggested models that detect stress automatically using conventional machine learning algorithms, some issues remain unsolved. One of these is the complexity of the classification steps. Four steps are involved in conventional methods: Preprocessing, feature selection, classifier training, and classification. Preprocessing, including R-peak extraction, is required to extract the proper R-peak and calculate the HRV. Most of these studies used other physiological signals (i.e., respiration, SC, and ST) as well as an ECG, and needed to select proper features among the both physiological signals and the HRV parameters. These processes make it difficult to apply these models in a practical environment, from the perspective of real-time classification. Additionally, a person must measure the ECG for at least 1 min to detect a stress state, even though it is a short-term HRV analysis.

In this paper, we suggest a DNN model to detect stress with a raw ECG signal, which possibly overcomes the limitations mentioned above. An end-to-end method using a raw ECG signal does not require preprocessing (i.e., filtering and R-peak extraction). Additionally, this method does not require additional feature selection. The other contribution of our research is a method for training the DNN with a pretrained model. The DNN requires a large amount of data to train the model, but it is difficult to acquire a large data set of physiological signals. We trained the proposed model based on a pretrained model, which learned a large amount of data [14]. We evaluated the performance of the proposed model by calculating evaluation metrics (e.g., accuracy and area under the curve) which are widely used in the evaluation of DNN models. We also assessed the proposed model by comparing it with conventional machine learning methods [9,10,11] which used only the ECG to detect stress, and with other DNN methods [12,13].

## 2. Material and Methods

### 2.1. Subjects and Data Acquisition

**Figure 1:**
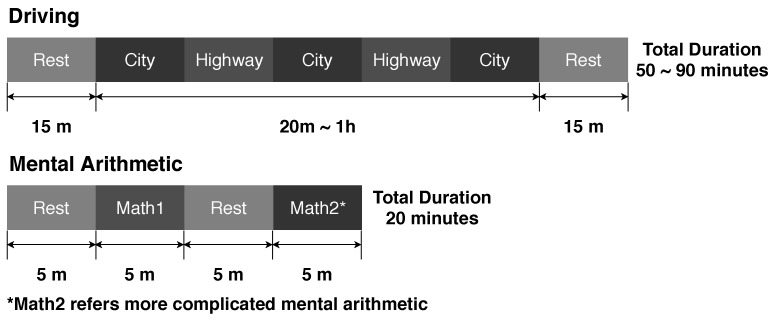
The experimental procedures and their durations.

We used two kinds of data sets, which were obtained from two different experiments, to train and evaluate the proposed model. The two different data sets can be considered as ambulatory and laboratory stress, respectively. One of the data sets consisted of ECG measurements collected from drivers who drove through a city and on a highway [14]. The other data set was recorded in an experimental environment, where mental stress was induced by arithmetic tasks in the participants. Figure 1 shows detailed information about both protocols, including duration and task.

#### 2.1.1. Driving Data Set

PhysioNet [23] offers free access to a large number of physiological signals recorded under various conditions. We selected the driver stress data set [14] from among the free accessible databases. It consists of various physiological signals, including ECG, electromyography (EMG), SC, and respiration signals, which were recorded under the conditions of driving and resting. Healey et al. [14] tried to monitor real-world stress during driving situations. Among the recorded physiological signals, we chose the ECG, which was sampled at 496 Hz. Modified lead II configuration electrodes were used to measure the ECG. Sixteen participant’s records were uploaded to PhysioNet. We excluded 2 subject’s data, as they did not contain a record of the marker for when the participants changed the driving region to highway or city. Finally, we selected 14 subject’s records for use in our study. All the selected records included approximately 50–85 min of ECG measurements during driving and resting. Due to differences in traffic conditions, the total duration of experiments differed by subject. The participants were made to take a rest for 15 min before and after driving.

#### 2.1.2. Mental Arithmetic Data Set

Seventeen people (6 female and 11 male, 27.9±3.3 years old) participated in our experiment. We designed an experiment to induce mental fatigue in the participants using a mental arithmetic task. The mathematical tests were developed based on the Montreal Imaging Stress Task (MIST) to elicit two different levels of mental stress in participants. To do so, we simplified the MIST paradigm by two levels of difficulty. The participants had to try to solve the arithmetic problems and push the keypad to answer the questions. The problem consists of two levels: Moderate and high. The moderate level included three integers with plus and minus operations. For the high level, four integers and all of the arithmetic operations were used. All participants encountered the same level of complexity for the arithmetic problems. The ECG data were sampled at 256 Hz and measured by a T-REX TR100A sensor; the electrodes were placed in a modified lead II configuration, which was the same as for the driving data set [14]. First, we measured the baseline ECG for 5 min while the participants took a rest. After the baseline measurement, the participants took the mental arithmetic test two times (5 min each) at a moderate and a high level. They encountered more complicated mathematical problems during the high level test. We provided a 5 min rest between the mental arithmetic tests. To measure whether the mental arithmetic induced mental stress in the participants, we used two questionnaires, including self-assessment manikin (SAM) [24] and distress thermometer (DT) [25]. The self-reports were written after the first rest period and after two repetitions of the tasks. The mental arithmetic experiments were approved by the institutional review board at the Korea Institute of Science and Technology (2017-030).

### 2.2. Data Preprocessing and Annotation Procedures

We performed some preprocessing procedures before using the data sets for training the neural network. As the ECGs of the two data sets were recorded by different sensors, we scaled them to the same range (0–1) using z-score normalization,

$$\begin{array}{c} x_{i}^{s}\leftarrow \frac{x_{i}^{s}-\mu^{s}}{\sigma^{s}},\;\mathrm{for}\;0\leq i<n\\ s\in \left\{\mathrm{Driving},\;\mathrm{MA}\right\} \end{array}$$

where *n* denotes the number of data sets for each stressor and window. We calculated the mean (μ) and standard deviation (σ), along with all the data for each stressor (i.e., the driving and the mental arithmetic). We normalized the ECG using both the mean and standard deviation. After normalization, the driving data set was downsampled to match the sampling rate of the mental arithmetic data set, which was sampled at 256 Hz. We needed a fixed input dimension, due to using the same neural network for training the model and detecting the stress based on the two different data sets. The ECG signals were sliced into 10 s, 30 s, and 60 s windows to detect stress in short-term windows. The reason for setting a short-term window was to try to recognize stress in nearly real-time.

We needed to annotate the data with specific labels to train a neural network by a supervised method. We segmented the driving data set, based on the boundaries between driving on the highway, driving on the city, and resting. The ECG measurements recorded during driving on the highways and in the cities were labeled as stress, and the other measurements were labeled as rest. In the case of the mental arithmetic data set, we labeled the ECG measurements recorded during the mathematical task as stress. The other ECG measurements, recorded during the rest period, were annotated as rest.

Table 2 shows the numbers of the data sets and their label distribution for each window. The number of data labeled as stress in the driving data set was much larger than those labeled as rest, while the mental arithmetic data set shows a balanced label distribution. The drivers took a rest for approximately 30 min, including an initial and final rest, but they drove for over 45 min, resulting in an imbalanced distribution. The participants who took the mental arithmetic test were exposed to the same amount of time for the stress task and resting; 10 min each. The driving data set and the mental arithmetic data set contained over 72,000 and 16,000 ECG cycles, respectively.

### 2.3. The Deep Neural Network

**Figure 2:**
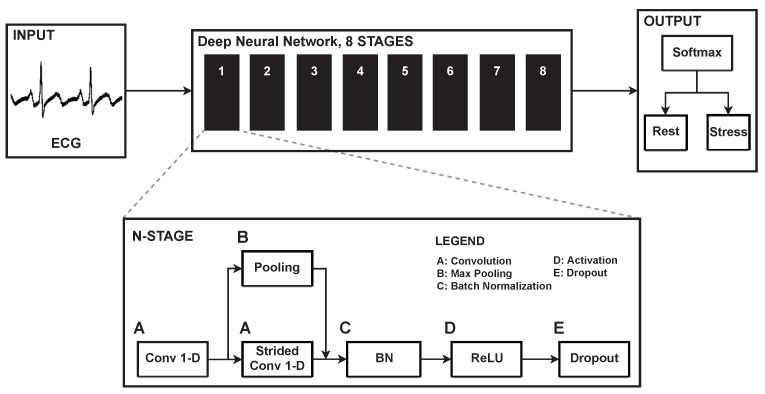
Deep neural network architecture and the components of each stage. Raw ECG signals are provided into the input layer. The successive stages extract features from an output of a previous stage. After the last stage, a softmax classifier performs a binary classification between the rest and stress.

We propose a deep convolutional neural network to detect stress events. Figure 2 shows the architecture of the proposed model. It obtains input of only raw ECG signals, not HRV parameters or other physiological signals. The input dimension can be defined as $x\in R^{m\times w\times c}$, where *m* and *c* denote the size of the mini-batch and the number of channels, respectively, and *w* refers to the width of the ECG, which is defined as the multiplication of the sampling rate and window. Our model contains successive stages (N=8) to extract features from the ECG. Table 3 shows the list of the operations and the detailed parameters used in the stages. The number of filters in the convolutional layer is defined as $8\times 2^{k}$, where *k* begins at 0 and is increased by one every second stage. Each stage consists of two convolutional layers and one pooling layer. A convolutional layer performs a convolution operation with its filter and a specific stride. A stride is defined as how much the filter moves within a layer (i.e., the convolutional and pooling layer). An output of the first convolutional layer is fed to both a strided convolutional layer and a pooling layer. The stride values of the strided convolutional layer and pooling layer are set to 2. The inputs of these two layers are subsampled by a factor of 2. Max-pooling, which chooses the maximum value among the filter widths, is used in the pooling layers. Both the strided convolutional layers and the pooling layers subsample their inputs, followed by each input being concatenated along its channels. If the output of a previous stage has a $\sigma^{\left(N-1\right)}\in R^{m\times w\times c}$ dimension, both the strided convolutional layer and pooling layer produce outputs as $\left\{C^{\left(N\right)},P^{\left(N\right)}\right\}\in R^{m\times (w/2)\times (c/2)}$, where $C^{\left(N\right)}$ and $P^{\left(N\right)}$ denote the output of the strided convolutional layer and pooling layer in the *N* stage. Concatenating along its channels gives it a dimension of $\sigma^{\left(N\right)}\in R^{m\times (w/2)\times c}$. When passing through the stages (N=1,2,⋯,8), dimension reduction along its width is performed. For example, if the input (raw ECG) has dimensions of $x\in R^{m\times w\times 1}$,

$$\begin{array}{cc} \sigma^{\left(N\right)}\in R^{m\times (w/2^{N})\times c,} & N\in \left\{1,2,\ldots ,8\right\}\\ w=256\times \mathrm{window}, & \mathrm{window}\in \left\{10,30,60\right\}\\ c=8\times 2^{k}, & k=\left\{\begin{array}{c} \frac{N}{2}-0.5,\;\mathrm{when}\;N\;\mathrm{is}\;\mathrm{odd}\\ \frac{N}{2}-1,\;\mathrm{when}\;N\;\mathrm{is}\;\mathrm{even} \end{array}\right. \end{array}$$

The extracted features ($\sigma^{\left(8\right)}$) generated by the last stage are fed to the softmax classifier, which performs a binary classification between stress and rest:

(1) $$h_{j}=\frac{\exp\left(\sigma_{j}^{\left(8\right)}\right)}{\sum_{k=1}^{2}\exp\left(\sigma_{k}^{\left(8\right)}\right)},\;j=1,2$$

where j=1,2 for the binary classification. The output of the softmax classifier, $h_{j}$, represents the probabilistic distributions of each class—stress and rest—the sum of which is 1. Table A1 shows more detailed information of the proposed network, including the shape of the output for each operation with the input of 10 s of ECG.

We used a rectified linear unit (ReLU) as an activation function to generate a non-linear decision boundary from the successive linear combinations of the weighted inputs. The activation function produces a maximum value between zero and its input. We applied dropout [26] with a drop rate of 0.3 and batch normalization [27] to prevent overfitting. A neural network can be easily overfitted to the training data when a model learns within a small number of data sets. Many studies have made use of dropout and batch normalization to overcome overfitting. Dropout requires a drop rate, which represents how many neurons are dropped in each layer. Batch normalization makes the input data follow a specific distribution, based on a normalized input distribution. The distribution can be changed during training through the trainable variables [27] γ and β, where γ scales the normalized input and β shifts it.

### 2.4. Training the Neural Network

There are three types of training method for the proposed model: Type I generates a pretrained model that trains using the driver data set; Type II trains a model with the mental arithmetic data set; and Type III trains a pretrained model (i.e., Type I) with the mental arithmetic data set.

All three types of training use the same end-to-end architecture, using the raw ECG signals from each data set. A loss function needs to be set to train the DNN model. We utilized the cross-entropy loss function:

(2) $$L=-\frac{1}{m}\sum_{i=1}^{m}\left(y_{i}\log\left(h_{i}\right)+\left(1-y_{i}\right)\log\left(1-h_{i}\right)\right),$$

where $h_{i}$ and $y_{i}$ denote the prediction results from the proposed model and the true labels from the data set, respectively, and *m* represents the size of the mini-batch, which is set to 64. When the model predicts a state (i.e., stress or rest) properly, the loss function becomes nearly zero. However, it diverges from zero in the opposite situation; that is, when the model produces an output different from the data set label. A proper optimizer must be selected to train the DNN stably, because the optimizer ensures that the loss function converges to zero. We used the Adam optimizer ($\beta_{1}\;=\;0.9,\beta_{2}\;=\;0.999,\epsilon \;=\;10^{-8}$) [28] to train the proposed model. This optimizer calculates the gradients of the loss function by back-propagation, which adjusts the weights of the neurons in an end-to-end model. All the weights of the neurons are initialized by the He initializer [29].

(3) W∼N(0,Var(W)).

The weight distribution is initialized to the normal distribution, which has a mean of zero and standard deviation of the weight variance. The weight variance is defined as follows:

(4) $$Var\left(W\right)=\sqrt{\frac{2}{n_{in}}},$$

where $n_{in}$ denotes the number of input weights.

There are many hyperparameters (e.g., the number of layers, number of neurons, size of the mini-batch, filter width, number of channels, among others) to be decided, in order to train a model properly. We first considered how many stages are adequate for the proposed model. The number of the stages began with 1, and the performance for accuracy showed improvement as the number of stages increased by 1, up until 8. Within the proper number of stages, we tuned the filter width and the number of filters to find the best fitting parameters. We searched the hyperparameters of the DNN through a grid search method and a manual search. Finally, we chose the model that achieved the highest accuracy for the test data set along all the maximum 10 epochs. Section 2.5.1 shows how we split the data set into a training set and testing set, based on cross-validation.

#### 2.4.1. Type I Training

We generated a pretrained model with the driving data set. As the DNN requires a large amount of data for training, we used the driving data set, which was larger than the mental arithmetic data set. The learning rate was set to $1\times 10^{-3}$ and was reduced by a factor of 10 every 5 epochs.

#### 2.4.2. Type II Training

Using the mental arithmetic data set, the same method of end-to-end training was applied as in Type I training. Additionally, we used all the same hyperparameters to observe how the size of the data set affected training the neural network.

#### 2.4.3. Type III Training

We applied a transfer learning method, using the pretrained model generated by Type I training. As mentioned above, it is difficult to train a neural network with a small data set. If a model is trained based on a pre-trained model, the model can then easily be fine-tuned. We hypothesized that the ECG measurements obtained from the participants who had taken the mental arithmetic task were similar to those obtained from drivers. It is effective to apply transfer learning when the distribution of data set to be learned is similar to that of the pretrained model. The softmax classifier required a re-training process, because there is little difference between the data used in pretraining and the data to be trained, although their distributions were similar. However, re-training only the softmax classifier did not show an acceptable performance. The number of layers to retrain was, thus, considered as a hyperparameter. We applied the grid search method to find the proper stages (Stage 1, Stage 2, …, Stage 8) to be retrained. The start stage to be retrained was changed until a satisfactory performance was achieved. We kept the pretrained model, except for the last stage (N=8) and the softmax layer (N=9). The trainable variables in the softmax classifier were initialized before training.

(5) $$W^{\left(N\right)}∼N\left(0,Var\left(W^{\left(N\right)}\right)\right).\;N=9\;\left(\mathrm{softmax}\right)$$

We utilized the Adam optimizer [28] to update the trainable variables of the last stages and softmax classifier through backpropagation, but the variables in the other stages were kept constant:

(6) $$W^{\left(N\right)}\leftarrow W^{\left(N\right)}-\alpha_{t}\cdot m_{t}/\left(\sqrt{v_{t}}+\epsilon \right),\;N=8,9$$

where *m* and *v* denote the first moment and second moment of the Adam optimizer, respectively. We used a different learning rate (α), which started at $1\times 10^{-4}$ with the same decay rate (decreasing by a factor of 10 every 5 epochs), to train the model. As the pretrained model was already fine-tuned, it was better to use a lower learning rate. The results of three training types and comparisons between them are shown in Section 3.

### 2.5. Model Evaluation

In this section, we describe how to evaluate the proposed model. All three training types, as mentioned above, performed training with a training set using cross-validation. We tested each type of end-to-end model with its test set and calculated the evaluation metrics (i.e., the receiver operating characteristic curves). Additionally, we observed the features not only at the end of the neural network, but also in the middle stages. The T-distributed stochastic neighbor embedding (t-SNE) [30] makes high-dimensional features visible in a two- or three-dimensional domain.

#### 2.5.1. Cross-Validation

We used k-fold cross-validation (k=10) to evaluate the proposed model. Both the driving and the mental arithmetic data sets were split by subject by cross-validation. The DNN should not have seen data in the test set presented during training. It is obvious that a neural network achieves a high performance with the data used in training. In other words, we needed to divide the data set into both a training set and a test set, and perform training and testing based on each set individually. In the case of the physiological signals, it is difficult to acquire a satisfactory amount of data to train a neural network. There are several limitations in a laboratory environment, such as the portability of the sensors and the inconvenience of the person to be measured. However, cross-validation makes it possible to generate both the training and testing sets with only a small amount of data. We randomly split the data set into individual subjects that make *k* folds using cross-validation. Each fold consists either of one subject or more than one subject. We trained the models with k−1 folds and assessed them with the one fold left. Thus, the training and test sets had a 9:1 ratio. All these processes were iterated *k* times with the individual end-to-end models, which produced *k* models. Therefore, each model was trained by an individual training set and also validated by a test set which had never been seen during training. All of the three training types were evaluated with the data set which was included in the same data set used during the training session, but the model never had seen it. For example, the Type I model was trained and tested with the driving data set. In the case of Type III, although the pretrained model was made based on the driving data set, it was retrained using the mental arithmetic data set. Therefore, the mental arithmetic data set was used for the evaluation. All the evaluation metrics were cross-validation results, which are the mean values of all the folds.

All training and validation was performed on a personal computer (CPU; AMD Ryzen 7 2700X, GPU; NVIDIA GeForce GTX 1080 Ti 11 Gb, Memory; 32 Gb). With the use of GPU, it took less than two and one seconds per epoch for training the model with the driving and mental arithmetic data sets, respectively. However, without GPU, the driving data set required 22 s per epoch and the mental arithmetic data set needed 5 s per epoch to train the model.

#### 2.5.2. Statistical Analysis

A softmax classifier placed at the end of the DNN produces probabilistic outputs, which indicate how likely it is that the inputs are related to the true labels. Among the outputs, a classifier selects the highest probability for its predictions. It is an important way to compare these predictions with the true labels to evaluate the model performance. Many metrics are used to assess such models, such as receiver operating characteristic (ROC) [31] curves and precision–recall (PR) curves [32]. An ROC curve plots sensitivity against 1-specificity with a changing threshold value. Similarly, the precision against the sensitivity (recall) is plotted in the PR curves, which gives an additional analysis to the ROC curves for an imbalanced data set [32]. We calculated the area under the curve (AUC) for the ROC curves, which was nearly 1.0 when the model had successfully operated. We also computed the $F_{1}$ score, which represents the mean of sensitivity and precision. The sensitivity, also called recall, refers to how well a model detects stress among the true stress events. The specificity shows the correct detection rate of the rest state. The precision represents the ratio of the number of true-positives to the number of cases in which a model predicted stress. We compared the proposed model to other models [9,10,11,12,13], and to itself, for each type of training (i.e., Types I, II, and III) using the evaluation metrics. We utilized one-way analysis of variance (ANOVA) and Tukey’s test to assess the model itself within each training type.

## 3. Results

We collected two self-reports (e.g., SAM and DT) from the participants after two levels of the mental arithmetic task and after the initial rest. Lower SAM and higher DT scores refer to stronger negative emotions and higher perceived stress, respectively. Table 4 shows the results of the self-reports. We calculated the difference of score based on thebaseline measurement (i.e., after initial rest) after the mental arithmetic task. SAM decreased after the tasks and the difference for the high level task was larger than that for the moderate level. The DT score increased, compared to the baseline measurement. Similar to the SAM score, a large difference in the DT score occurred after the high level arithmetic task.

In this section, we show the results of the proposed model. It consists of the extracted feature maps and evaluation metrics, including a comparison with the other models and within the proposed model itself. As mentioned in Section 2.4, Type I training indicates the pretrained model using the driving data set. For Type II, the model was trained using the mental arithmetic data set without the pretrained model. In the case of Type III training, we used the same data set as in Type II to train the model, but based it on the pretrained model.

Firstly, we tested the conventional machine learning methods before evaluating the proposed model. We used conventional algorithms, including decision tree (DT), k-nearest neighbors (kNN), logistic regression (LR), random forest (RF), and support vector machine (SVM). All the algorithms were trained and validated with the same data set as the proposed model. Table 5 shows the accuracy of the conventional methods. All the machine learning algorithms could not reach a satisfying performance, in terms of accuracy, which means that trainable algorithms cannot learn the proper features using a raw ECG input.

### 3.1. Feature Representation

**Figure 3:**
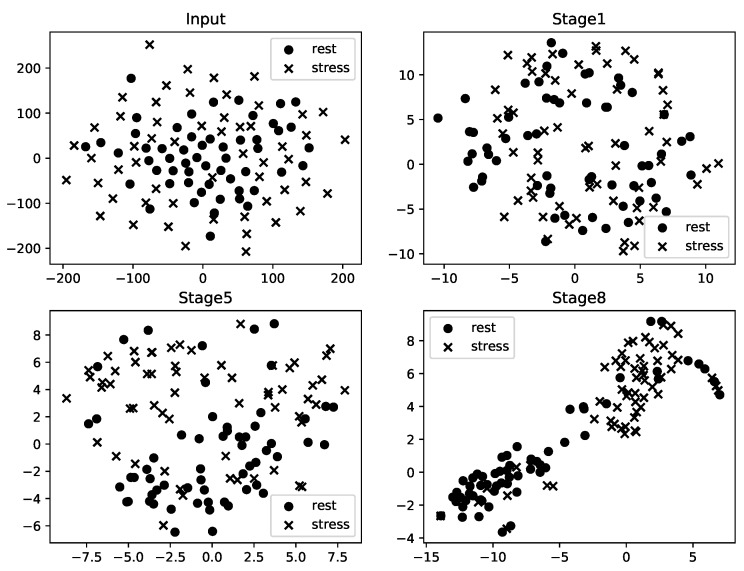
The t-SNE plots of raw ECG and extracted features from the stages. Round points denote features of ECG labeled as rest, and crosses represent stress-labeled features. This figure shows only the extracted features from stage 1, stage 5, and the last stage.

We observed all the extracted features from each stage using the t-SNE method, which converts high-dimensional features (the number of components, width, and channel) to 2-dimensional features, which we can analyze using a scatter plot. Figure 3 shows the t-SNE scatter plots for the input (raw ECG) and the extracted features from each stage. Each point represents states of the label (i.e., rest and stress). The input is from a subject who participated in the mental arithmetic task and is sliced using a 10 s window. The proposed model, trained by Type III training, generated features in each stage. As shown in Figure 3, there was almost no difference between the stress- and rest-labeled ECGs. By considering the features passed through the stages, a distinction could be observed between the labels. The t-SNE plots imply that it is possible to distinguish the two labels clearly through the softmax classifier after the last stage.

### 3.2. Performance of the End-to-End Model

**Figure 4:**
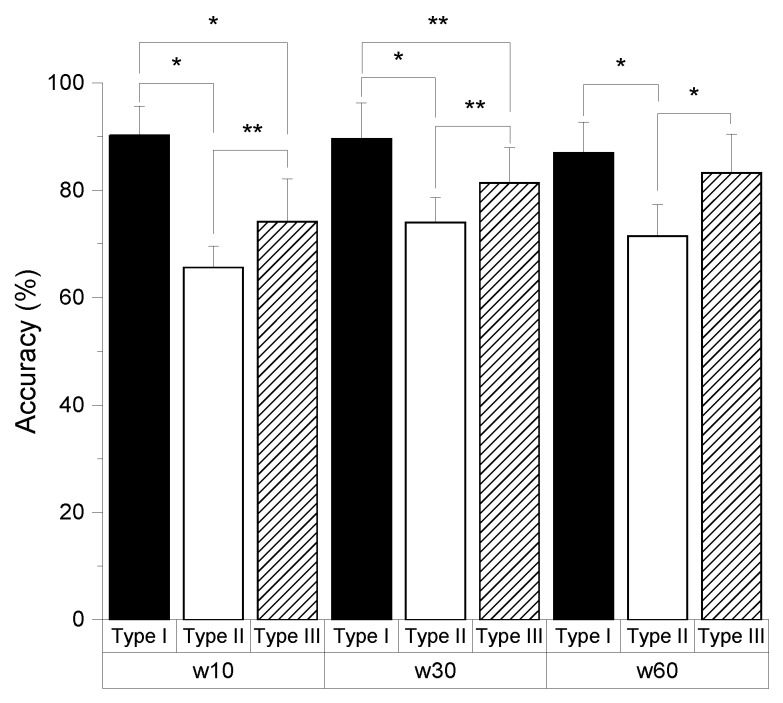
Accuracy of the end-to-end model in binary classification. Types are grouped by each raw ECG window (i.e., 10 s, 30 s, and 60 s) fed to the model. * and ** indicates that difference of the means is significant at the 0.001 and 0.05 level, respectively.

Figure 4 shows the accuracy of the proposed model for the binary classification of rest and stress. We compared the results of the three training types, based on the input windows. Overall, Type I, which was trained using the driving data set, showed the best performance for all the windows. It reached the highest mean accuracies at 89.38%, 87.16%, and 79.12% for the 10 s, 30 s, and 60 s windows, respectively. The accuracy of Type I training, 89.38%, was significantly different from both the Type II accuracy, 61.33%, and Type III accuracy, 69.71%, for the 10 s window (p<0.001). Additionally, there was a significant difference between the accuracy of Type II and Type III training (p<0.05). In the case of the 30 s window, Type I training achieved an accuracy of 87.16%, whereas Type II and Type III training achieved 68.38% and 72.13%, respectively (p<0.001). For the 60 s window, the accuracies of Type I, 79.12%, and Type III, 79.50%, training were slightly different, but the accuracy of Type II training, 71.50%, was significantly different from that of Type III training (p<0.05). Considering Type II and Type III training, which were both trained with the same data set (mental arithmetic), there were improvements of 12.01% and 10.06% in accuracy for the 10 s and 60 s windows (p<0.05), respectively; while there was no significant improvement in the 30 s window at the 0.05 level.

**Figure 5:**
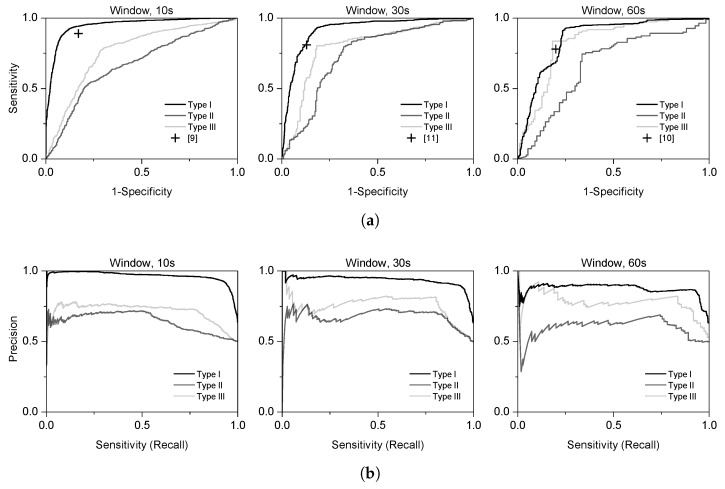
ROC and PR curves. Each line represents a curve from Type I, Type II, and Type III training, respectively. A cross refers to the performances of the conventional model. (**a**) ROC curves and (**b**) PR curves.

We plotted the ROC and PR curves by each type and window in Figure 5. The ROC curves need to be located above the baseline (y=x) to satisfy the model performance. We can observe that Type I training showed the best performance in the ROC and PR curves. Type III training demonstrated little improvement over Type II training, based on the curves. However, both the ROC and PR curves of Type III training are generally positioned higher than the curves of Type II. It is difficult to evaluate the performance of the model with the ROC and PR curves only. Therefore, we calculated the AUC of the ROC curves. It shows the performance on the numerical results, which makes it possible to compare the models.

Table 6 shows the evaluation metrics, including the AUC, $F_{1}$ score, sensitivity, and specificity. Both the mean and standard deviation were calculated based on cross-validation. We compared the performance between Type I and Type II training, which were both trained without any pretrained model, but trained with the different sizes of data sets (i.e., the driving and the mental arithmetic data sets). Type I training for the 10 s window shows the best performance for the AUC, $F_{1}$ score, sensitivity, and specificity. It had a value of 0.938 for the AUC (p<0.001), 0.922 for the $F_{1}$ score (p<0.001), and 0.930 for sensitivity (p<0.001). Although the specificity of Type I training for the 10 s window, 0.854, showed the highest value, it did not show a significant difference from Type II training. Based on the mean values, Type III training showed an improvement over Type II training, except for specificity with the 10 s window. For the 10 s window, the improvements were 8.00% for the AUC, 19.90% for the $F_{1}$ score (p<0.001), and 29.77% for sensitivity (p<0.05). For the 30 s window, the improvements were 5.07% for the AUC, 7.42% for the $F_{1}$ score, 1.81% for sensitivity, and 16.61% for specificity. The 60 s window showed improvements of 18.66% for the AUC (p<0.05), 13.23% for the $F_{1}$ score (p<0.05), 7.32% for sensitivity, and 20.71% for specificity. In summary, the transfer learning method improved performances by 11.57%, 10.57%, 13.52%, 12.96%, and 9.41% on average for accuracy, the AUC, the $F_{1}$ score, sensitivity, and specificity, respectively, along all window lengths.

### 3.3. Comparison with Different Models

We compared the proposed end-to-end model with conventional methods [9,10,11]. Rigas et al. [9] used physiological signals including HRV, SC, and respiration while using 10 s length of window. Smets et al. [11] additionally utilized skin temperature. Castaldo et al. [10] used non-linear HRV parameters, including the sample entropy (SampEn), recurrence plot mean line length (RPlmean), and shannon entropy (ShanEn). Figure 5a shows the comparison results to the proposed model using the ROC curves. Each blue cross is positioned at the best performance in [9,10,11]. To assess the model exactly, we compared it with [9,11], which used 10 s and 30 s windows, to the proposed model with the same window lengths. To best match Castaldo et al. [10], which used a 3 m window to extract the HRVs from the ECG, we compared the proposed model with the 1 m window. Based on Figure 5a, all blue crosses are positioned lower than Type I, or are similar to it. From the perspective of sensitivity and specificity, the proposed model shows better performance than the conventional methods for a certain range of thresholds.

Both Hwang et al. [12] and Saeed et al. [13] utilized DNNs to classify stress. The comparison results are shown in Table 7. Hwang et al. [12] used a CNN and LSTM with a raw ECG signal and achieved an 87.39% and 73.96% accuracy for each case. Their architecture consisted of one convolutional layer and two LSTM layers. Our proposed model shows improvements in accuracy of 3.10% and 18.00% for each case, with the same window (10 s). Saeed et al. [13] used raw HR signals derived from the ECG and raw SC signals. They used the same driving data set from Healey et al. [14] to train and evaluate their model. The model [13] showed the best performance, with a value of 0.918 for the area under the ROC curve, while the proposed model reached 0.938.

## 4. Discussion and Conclusions

We have proposed a novel end-to-end architecture that uses raw ECG signals for stress detection and validated its performance with two different data sets. We believe that our model could replace the conventional machine learning-based methods in several ways. First, in terms of model simplicity, our model has an advantage over conventional methods, which require a few additional steps, such as preprocessing, feature selection, and feature extraction, before classification. As our model was built with an end-to-end architecture, it does not necessarily require such additional steps. The end-to-end architecture enables the detection of stress by automatically extracting features without feature selection. We observed that the successive deep convolutional layers extract distinguishable features, as shown in Figure 3. Second, in the same vein, our model may not depend on the performance of these steps. The methods that use HRV parameters depend highly on the performance of the R-peak detection algorithm. Considering stress management in daily life, R-peak detection in ECG signals recorded in real-world environments may require additional steps, as proposed in [33]. In addition to the independence of the model, our results showed that the detection performance of the proposed model was superior to that of the conventional methods [9,10,11], as shown in Figure 5a and Table 5. With raw ECG signals, conventional machine learning methods did not show acceptable performance in detecting stress, and rarely can be trained with non-linear inputs (i.e., raw signals). Finally, whereas the HRV parameters require at least a short-term (5 m) or long-term window (24 h) to properly reflect the stress response, our model used much shorter windows (10 s, 30 s, and 60 s). Our approach demonstrates a practically applicable system for daily stress management. As it takes an average of 2.490 ms to estimate stress state by inputs of raw ECGs, it is possible to apply the proposed model in the real-world to detect stress in real-time.

Despite the advantages described above, the performance of the DNN depends highly on the size of the data set used to train a neural network. To investigate the effect of the data set size on stress detection, we compared three different types of models with different training strategies. As expected, the model Type I, which was trained using a larger data set, showed better performance than the model Type II, trained using a smaller data set. There was a size difference of more than four times between the driving [14] and the mental arithmetic data sets. For the last type of model (Type III), we utilized the pretrained model, trained using the driving data set, to train the model with a smaller data set. From the comparison between Type II and Type III training, Type III, which used the pretrained model, showed an improvement over Type II, which did not. Although the size of the mental arithmetic data set might not be large enough to train the neural network, it is possible to achieve a fine-tuned model, based on pretraining with a larger data set. However, it could not reach the performance of the Type I model, trained using a larger data set, which presented the best performance. Unlike other domains of data, such as speech or image, a sufficient amount of physiological data may not be easily accessed or obtained. Thus, our approach can be utilized to train a DNN with a smaller data set, based on the pretrained model.

In this study, we used two different data sets (i.e., driving and mental arithmetic) under the ambulatory and laboratory environments for model development and validation. Mental arithmetic is one of the representative test paradigms used to assess mental stress. It was proved, by two questionnaires (self-assessment manikin and distress thermometer), that mental arithmetic induced a mental load in the participants. However, to develop a stress management method for daily life, there is a need to validate the method out-of-laboratory, as well. Thus, we also chose the driving data set to assess stress out-of-laboratory. Although these data sets cannot represent all of the stress situations that can occur in everyday life, such as workload stress, physical stress, anxiety, and so on, we demonstrated an end-to-end architecture to detect mental stress for both in- and out-of-laboratory environments. However, there were still limitations in this study. Although the two sensors used in the two data sets were individual, in view of generalization, the model needs to be validated by using ECG from diverse sensors, including other electrode configurations. We have fed other data sets, which were different from those using during training, into the model. This showed high-biased results about a specific type of stress and recording sensor dependency. Even though bias or dependency remains, transfer learning from one data set to other may provide a solution to break the limited applicability in real-world settings. As mentioned above, all of the data sets used in this study were acquired during specific stressful tasks. However, ECGs during daily activities are necessary for considering daily monitoring of stress. In future studies, we will apply this model to detect other stressful events, such as workload stress or anxiety, and will apply it to multi-class problems or continuous level recognition. Additionally, we will investigate how to augment physiological signals to train a neural network to overcome the limitations of the data set.

## Figures and Tables

**Table 1:** Previous studies on stress detection. ECG, Electrocardiogram; SC, Skin Conductance; ST, Skin Temperature; HR, Heart Rate; BN, Bayesian Network; AB, Adaptive Boosting; CNN, Convolutional Neural Network; RNN, Recurrent Neural Network; MT-CNN, Multitask CNN; AUC, Area Under the Curve; SCWT, Stroop Color Word Test; MA, Mental Arithmetic.

Ref	# of Subjects	Signal	Window Length (s)	Classifier	Performance (%)	Stressor
[9]	13	ECG, SC, Respiration	10	BN	84	Driving
[10]	42	ECG	180	AB	80	Verbal examination
[11]	20	ECG, SC, Respiration, ST	30	BN	84.6	SCWT, math, counting
[12]	20, 30	Raw ECG	10	CNN+RNN	87.39, 73.96	MA, interview, SCWT, visual stimuli, cold pressor
[13]	10	HR, SC	-	MT-CNN	0.918 (AUC)	Driving [14]

**Table 2:** Number of samples in the data sets.

Stressor	Window Length (s)	Number of Samples		Total
		Rest	Stress	
Driving	10	2161	3731	5892
	30	712	1227	1939
	60	349	598	947
Mental arithmetic	10	1020	1020	2040
	30	340	340	680
	60	170	170	340

**Table 3:** List of operations and hyperparameters used in each stage.

Order	Operation	Filter Width	Number of Filters	Stride
1	Conv 1-D	16	$8\times 2^{k}$	1
2	Conv 1-D	16	$8\times 2^{k}$	2
	Pooling	16	-	2
3	Concat	Concatenating		
4	BN	Batch normalization		
5	Activation	ReLU		
6	Dropout	Drop rate: 0.3		

**Table 4:** Difference in self-reported scores, compared to baseline measurement.

Task	SAM	DT
Math1	−0.37	0.37
Math2	−0.58	0.89

**Table 5:** Accuracy of the conventional methods. (DT; Decision Tree, kNN; k-Nearest Neighbors, LF; Logistic Regression, RF; Random Forest, SVM; Support Vector Machine).

Stressor	Classifier	Window Length (s)		
		10	30	60
MA	DT	0.539 (0.050)	0.517 (0.062)	0.490 (0.066)
	kNN	0.497 (0.030)	0.511 (0.040)	0.535 (0.058)
	LR	0.493 (0.029)	0.537 (0.076)	0.508 (0.055)
	RF	0.512 (0.075)	0.505 (0.062)	0.515 (0.041)
	SVM	0.483 (0.025)	0.516 (0.071)	0.520 (0.082)
Driving	DT	0.487 (0.210)	0.457 (0.234)	0.512 (0.208)
	kNN	0.361 (0.051)	0.423 (0.150)	0.451 (0.208)
	LR	0.447 (0.188)	0.443 (0.235)	0.434 (0.225)
	RF	0.528 (0.187)	0.486 (0.215)	0.523 (0.193)
	SVM	0.514 (0.155)	0.533 (0.177)	0.498 (0.205)

**Table 6:** Evaluation metrics.

Type	Window Length (s)	Evaluation Metrics			
		AUC	$F_{1}$ Score	Sensitivity	Specificity
I	10	0.938 (0.053)	0.922 (0.044)	0.930 (0.035)	0.854 (0.094)
II		0.701 (0.069)	0.602 (0.094)	0.552 (0.186)	0.759 (0.173)
III		0.761 (0.088)	0.752 (0.079)	0.787 (0.117)	0.696 (0.144)
I	30	0.924 (0.072)	0.922 (0.050)	0.949 (0.039)	0.788 (0.161)
II		0.766 (0.049)	0.755 (0.050)	0.815 (0.143)	0.665 (0.165)
III		0.807 (0.131)	0.815 (0.063)	0.830 (0.130)	0.797 (0.170)
I	60	0.857 (0.141)	0.901 (0.036)	0.923 (0.044)	0.755 (0.214)
II		0.679 (0.113)	0.717 (0.078)	0.760 (0.227)	0.670 (0.258)
III		0.835 (0.095)	0.826 (0.089)	0.820 (0.162)	0.845 (0.161)

**Table 7:** Comparison with models featuring a DNN algorithm.

	[12]	[13]	Proposed
Window	10 s	-	10 s
Input	Raw ECG	Raw HR and SC	Raw ECG
Accuracy	87.39%, 73.96%	-	90.19%
AUC	-	0.918	0.938

## Appendix A

**Table A1:** More detailed information about the proposed architecture. “conv” denotes “conv(filter width)-(filter channel)”. Similar to “conv”, “maxpool16” refers to max pooling with 16 lengths of the filter.

Order	Operation	Output	Stride	# of Parameters
0	input	(?, 2560, 1)	-	-
1-1	conv16-8	(?, 2560, 8)	1	128
1-2	conv16-8	(?, 1280, 8)	2	1024
1-2	maxpool16	(?, 1280, 8)	2	-
1-3	concatenating	(?, 1280, 16)	-	-
1-4	batch normalization	(?, 1280, 16)	-	32
1-5	activation & dropout	(?, 1280, 16)	-	-
2-1	conv16-8	(?, 1280, 8)	1	2048
2-2	conv16-8	(?, 640, 8)	2	1024
2-2	maxpool16	(?, 640, 8)	2	-
2-3	concatenating	(?, 640, 16)	-	-
2-4	batch normalization	(?, 640, 16)	-	32
2-5	activation & dropout	(?, 640, 16)	-	-
3-1	conv16-16	(?, 640, 16)	1	4096
3-2	conv16-16	(?, 320, 16)	2	4096
3-2	maxpool16	(?, 320, 16)	2	-
3-3	concatenating	(?, 320, 32)	-	-
3-4	batch normalization	(?, 320, 32)	-	64
3-5	activation & dropout	(?, 320, 32)	-	-
4-1	conv16-16	(?, 320, 16)	1	8192
4-2	conv16-16	(?, 160, 16)	2	4096
4-2	maxpool16	(?, 160, 16)	2	-
4-3	concatenating	(?, 160, 32)	-	-
4-4	batch normalization	(?, 160, 32)	-	64
4-5	activation & dropout	(?, 160, 32)	-	-
5-1	conv16-32	(?, 160, 32)	1	16,384
5-2	conv16-32	(?, 80, 32)	2	16,384
5-2	maxpool16	(?, 80, 32)	2	-
5-3	concatenating	(?, 80, 64)	-	-
5-4	batch normalization	(?, 80, 64)	-	128
5-5	activation & dropout	(?, 80, 64)	-	-
6-1	conv16-32	(?, 80, 32)	1	32,768
6-2	conv16-32	(?, 40, 32)	2	16,384
6-2	maxpool16	(?, 40, 32)	2	-
6-3	concatenating	(?, 40, 64)	-	-
6-4	batch normalization	(?, 40, 64)	-	128
6-5	activation & dropout	(?, 40, 64)	-	-
7-1	conv16-64	(?, 40, 64)	1	65,536
7-2	conv16-64	(?, 20, 64)	2	65,536
7-2	maxpool16	(?, 20, 64)	2	-
7-3	concatenating	(?, 20, 128)	-	-
7-4	batch normalization	(?, 20, 128)	-	256
7-5	activation & dropout	(?, 20, 128)	-	-
8-1	conv16-64	(?, 20, 64)	1	131,072
8-2	conv16-64	(?, 10, 64)	2	65,536
8-2	maxpool16	(?, 10, 64)	2	-
8-3	concatenating	(?, 10, 128)	-	-
8-4	batch normalization	(?, 10, 128)	-	256
8-5	activation & dropout	(?, 10, 128)	-	-
	Total			435K
